# Breast Cancer Cell Membrane Camouflaged Lipid Nanoparticles for Tumor-Targeted NIR-II Phototheranostics

**DOI:** 10.3390/pharmaceutics14071367

**Published:** 2022-06-28

**Authors:** Mengze Xu, Yu Yang, Zhen Yuan

**Affiliations:** 1 Cancer Center, Faculty of Health Sciences, University of Macau, Macau 999078, China; yb97658@um.edu.mo; 2 Centre for Cognitive and Brain Sciences, University of Macau, Macau 999078, China; 3 Institute of Molecular Medicine (IMM), Renji Hospital, School of Medicine, Shanghai Jiao Tong University, Shanghai 200240, China; yuyang@shsmu.edu.cn

**Keywords:** NIR-II, photoacoustic imaging, photothermal therapy, homologous targeting, IR1048, cell membrane-camouflaged nanoparticles

## Abstract

Photoacoustic imaging and photothermal therapy that employ organic dye in the second near-infrared window (NIR-II) became an attractive theranostical strategy for eliminating solid tumors, in which IR1048 was previously reported to be a good candidate. However, the further biomedical application of IR1048 was blocked by its poor water-solubility and lack of tumor-targeting. To solve this problem, liposome camouflaged with 4T1 cell membrane fragments was employed to encapsulate IR1048 (thereafter called MLI), and its application for photoacoustic and thermo-imaging and photothermal therapy were explored in vitro and in vivo. The results showed that MLI exhibited spherical morphology around 92.55 ± 5.41 nm coated by monolayer adventitial fragments, and uniformly dispersed in PBS with high loading efficiency and encapsulation efficiency to IR1048. In addition, both free IR1048 and MLI presented strong absorption in NIR-II, and upon 1064 nm laser irradiation the MLI showed awesome photothermal performance that could rapidly elevate the temperature to 50.9 °C in 6 min. Simultaneously, phantom assay proved that MLI could dramatically enhance the photoacoustic amplitudes by a linear concentration-dependent way. Moreover, either flow cytometry or confocal analysis evidenced that MLI was the most uptaked by 4T1 cells among other melanoma B16 cells and Hek293 cells and coexist of IR1048 and 1064 nm laser irradiation were indispensable for the photothermal cytotoxicity of MLI that specifically killed 96.16% of 4T1 cells far outweigh the B16 cells while hardly toxic to the Hek293 normal cells. Furthermore, PA imaging figured out that 4 h post tail-vein injection of MLI was the best time to give 1064 nm irradiation to conduct the photothermal therapy when the average tumor-accumulation of MLI achieved the highest. In the NIR-II photothermal therapy, MLI could significantly inhibit the tumor growth and almost ablated the tumors with slight body weight variation and the highest average life span over the therapy episode and caused no damage to the normal organs. Hence, MLI could pave the way for further biomedical applications of IR-1048 by homologous tumor-targeting and dual-modal imaging directed NIR-II accurate photothermal therapy with high efficacy and fine biosafety.

## 1. Introduction

Female breast cancer is the most commonly diagnosed cancer and has caused an exceptionally high mortality worldwide; as such, it is a burning issue to be solved by advanced and legitimate forms of medicine [1]. Especially, the triple negative breast cancer with all negative expression of estrogen receptor, progesterone receptor and human epidermal growth factor receptor-2 figured prominently in its mortality and does not always respond to the majority of chemotherapeutic drugs [2,3]. Although as the first-choice management modality clinically [4], surgical resection of the breast decreased patients’ life-quality, and the poor prognosis and hard-to-reach residual tumor cells made it prone to relapse. Another commonly used means-radiotherapy was detrimental to patients due to frequent radiation damage [5]. Hence, people turned their eyes to emerging therapeutic methods such as multifunctional intelligent nanomedicine with the development of nanotechnology, biomaterials and bioimaging methods [6,7]. What is more, in the past four decades, tremendous efforts have been devoted to developing novel treatment methods such as targeted therapy, immunotherapy and photothermal therapy based on nanomedicine [8,9,10], among which the photothermal therapy that could convert the absorbed laser into heat to ablate solid tumors was evidenced as a powerful anticancer means with high selectivity. However, the conventional photothermal therapy is still limited by non-targeted toxicity and restricted biological tissue-penetration depth [11]. Additionally, conventional targeted strategies need much work to artificially modify the nanoparticles with ligands to simulate the binding to the receptors overexpressed on the surface of cancer cell membranes. In comparison, cancer cell membrane as natural targeted-head of nanoparticles was evidenced superior to achieve tumor targeting due to full reservation of membrane pathophysiological characteristic [12,13,14]. On the other hand, extensive reports unfolded that the penetration depth of near-infrared the first window (NIR-I, 750–900 nm) was still not enough for penetrate solid tumors due to its high tissue scattering [15,16,17]. Accordingly, the laser in the second near-infrared region (NIR-II, 1000–1700 nm) allowed for deeper tissue penetration, higher upper limit of radiation and greater tissue tolerance than that of the NIR-I showed greater application potential in photothermal therapy [18]. Moreover, imaging guided cancer therapy has been one of the representative therapeutic regimes [19]. Additionally, theranostical agents that could concurrently diagnose and treat malignant tumors were considered as the next generation of cancer therapy [20].

For example, photoacoustic imaging directed photothermal therapy was increasingly applied, because NIR-II phototheranostics attracted extensive attention in recent years by virtue of fine biological tissue penetration and high selectivity [21,22,23,24]. Whereas current single imaging method was not perfect to direct precise photothermal therapy, consequently multimodal imaging was introduced such as photoacoustic imaging, computed tomography and thermal imaging. Especially, photoacoustic imaging, a novel noninvasive and nonionic biomedical imaging method, can be utilized to monitor the therapeutic response of photothermal therapy due to its much higher in vivo imaging depth than conventional optical imaging and higher resolution and contrast than magnetic resonance spectrum and computed tomography [25,26,27].

Generally, theranostical agents were composed of inorganic nanomaterials such as gadolinium, gold, CuS or carbon nanotubes, or organic dyes (e.g., ICG and IR-1048), and co-delivered chemical drugs or proteins, genes, etc. However, inorganic nanomaterials were hindered for further in vivo biomedical application because of non-degradation and potential metabolism problems [28]. Thus, organic dyes delivery systems that could serve for both photothermal therapy and imaging simultaneously were highly desirable, in which IR-1048 was a potential candidate [19,29]. Nevertheless, IR-1048 was hydrophobic and devoid of tumor targeting. Therefore, to solve its problem and make full use of its anticancer function, we designed a breast cancer 4T1 cell membrane camouflaged lipid nanoparticles for breast tumor-targeted NIR-II phototheranostics. Because it has been preclinically and clinically evidenced that it will be more stable and helpful for efficacy enhancing and toxicity reducing to be encapsulated by liposomes, which is propitious to accumulated in tumor tissues by membrane fusion [30,31,32,33,34,35,36]. Furthermore, cancer cell membranes disguised liposomes could exert “self-recognition” interactions for homotypic targeting providing enhanced endocytosis [37,38,39,40]. As a consequence, breast cancer 4T1 cells membrane coated liposomes were employed to encapsulate IR1048 (thereafter called MLI) to improve its poor water-solubility and NIR-II photothermal efficacy.

In this study, MLI was prepared by film dispersion and membrane extrusion method, and their morphology and particle size were characterized by transmission electron microscopy (TEM) and dynamic light scattering (DLS) measurements. On the other hand, SDS-PAGE analysis was performed to analyze the protein profile of MLI, and its stability in PBS or FBS had been tested in 7 days. Secondly, the UV–vis–NIR absorption spectra showed that MLI also presented strong absorption peak in the second near-infrared window, for which 1064 nm was selected to irradicate its solution to explore its photothermal performance that recorded and photoacoustic behaviors. Thirdly, fluorescent labeling, flow cytometry analysis and laser scanning confocal techniques were used to investigate its homologous-targeting and cellular uptake. In addition, control variates method was introduced to the CCK-8 assay to assess the cell viability of MLI to 4T1, B16 and Hek293 cells. At last, 4T1 tumor-bearing mice model was established to evaluate its dual-modal imaging directed efficacy in vivo and biosafety.

## 2. Materials and Methods

### 2.1. Materials

1,2-distearoyl-sn-glycero-3-phosphoethanolamine-*N*-[(polyethylene glycol)-1000] (DSPE-PEG_1k_), 1,2-dihexadecanoyl-rac-glycero-3-phosphocholine (DPPC) and cholesterol were purchased from Avanti Polar Lipids Inc. (Birmingham, AL, USA), IR1048 dye was bought from Sigma-Aldrich (Darmstadt, Germany), while a membrane protein extraction kit was ordered from Beyotime Biotechnology. In addition, 1,1-dioctadecyl-3,3,3,3-tetramethylindocarbocyanine perchlorate (DiI) was ordered from YEASEN Biotechnology (Shanghai) Co., Ltd. (Shanghai, China). The utilized consumables including radio-immunoprecipitation assay (RIPA) lysis buffer, phenylmethanesulfonyl fluoride (PMSF), phosphatase inhibitors, 3,3′-dioctadecyloxacarbocyanine perchlorate (DiO) and enhanced cell counting Kit-8 (CCK-8) were purchased from Beyotime Biotechnology. Further, hoechst 33342 was purchased from US EVERBRIGHT, Suzhou, China. Fluorescein isothiocyanate (FITC) and Rhodamine B were ordered from Shanghai Yuanye Bio-Technology Co., Ltd. (Shanghai, China). Trypsin was bought from Gibco (Waltham, MA, USA). Calcein-AM and Propidium Iodide were ordered from Invitrogen (Waltham, MA, USA). In addition, all the chemicals used were not further purified. What is more, pure water used here was Milli-Q of 18.25 MΩ·cm (23 °C, EMD Millipore, Burlington, MA, USA).

### 2.2. Cell Culture and Animal Model

4T1 cells (mouse breast cancer cells) was cultured in Roswell Park Memorial Institute medium (RPMI-1640) supplemented with 10% fetal bovine serum (FBS, Gibco, USA) and 1% penicillin-streptomycin (PS, Gibco, USA), B16 and Hek293 cells were cultured with Dulbecco’s modified Eagle’s medium (DMEM) medium (Gibco, USA) containing 10% FBS and 1% PS in an incubator under 5% CO_2_ and constant temperature at 37 °C. All the animal experiments protocols were approved by the Animal Management and Ethics Committee of the University of Macau. Female BALB/c nude mice (4–6 weeks old) were supplied by the Shanghai Slac Laboratory Animal Co. Ltd. (Shanghai, China). 4T1 cells (5 × 10^6^) in 100 μL PBS were subcutaneously injected into the right legs of each mouse two weeks before conducting the tumor treatment. Tumor-bearing mice were used for dual modal photoacoustic and thermal imaging and PTT when the tumor size was up to 80~140 mm^3^. The tumor-bearing mice were randomly divided into six various treatment groups (n = 5): (1) PBS; (2) MLI; (3) Laser; (4) IR1048 + Laser; (5) LI + Laser; (6) MLI + Laser (1064 nm, 1 W/cm^2^ for 6 min).

Tumor volume = (tumor length) × (tumor width)^2^/2

### 2.3. Preparation and Characterization of Cell Membrane-Coated Liposomes Encapsulating IR1048 Nanoparticles (MLI)

The 4T1 cell membrane fragments were extracted from 4T1 cells. First, the logarithmic-phase 4T1 cells (3.2 × 10^7^) were pre-cooled on ice box for 6 min, and harvested gently by a rubber scraper into a 20 mL hypotonic solution (1 mmol L^−1^ NaHCO_3_, 0.2 mmol L^−1^ EDTA, and 1 mmol L^−1^ PMSF) at 4 °C overnight. Then, the swelling-ruptured cell suspension was grinded 20 times by using a Dons grinder, which was centrifuged at 4500× *g* for 5 min at 4 °C, firstly, and the supernatant was collected and centrifuged at 15,000× *g* for 30 min at 4 °C. The acquired pellet was resuspended with 1× phosphate buffered saline (PBS) and further sonicated with water-bath ultrasound for later use. 

The 4T1 cell membrane camouflaged IR1048-loaded liposomes were formulated through thin-film dispersion method and extrusion process. Briefly, the DPPC, DSPE-PEG1k and cholesterol was mixed into lipids with a molar ratio of 10:5:1, which then mixed with IR-1048 with 5:1 (*w/w*) and removed chloroform by co-evaporating method. In addition, the lipid film was hydrated by extracted 4T1 cell membrane fragments with a weight ratio of 1:1. The resulting solution was frozen for 10 min, and then thawed for 10 min, which was repeated for five cycles till extruded through a porous polycarbonate membrane of 100 nm by Avanti Miniextruder. In addition, the unencapsulated IR1048 were removed by a dialysis means (MWCO = 3500) and filtration through a 0.22 μm filter. Afterwards, its particle size and ζ potential were detected by Zetasizer NanoZS (Malvern, UK) and transmission electron microscope (TEM). The measurement of absorption spectrum was performed with a UV-is 1700 spectrophotometer. IR1048-loaded liposomes (LI) and blank liposomes were prepared and characterized with the same procedures. The loading efficiency (LE) and encapsulation efficiency (EE) of IR1048 were calculated,

$$\mathrm{LE}\left(\%\right)=\frac{\mathrm{weight}\;\mathrm{of}\;\mathrm{IR}1048\;\mathrm{in}\;\mathrm{MLI}}{\mathrm{weight}\;\mathrm{of}\;\mathrm{feeding}\;\mathrm{IR}1048+\mathrm{weight}\;\mathrm{of}\;\mathrm{feeding}\;\mathrm{materials}\;}\times 100\%$$

$$\mathrm{EE}\left(\%\right)=\frac{\mathrm{weight}\;\mathrm{of}\;\mathrm{IR}1048\;\mathrm{in}\;\mathrm{MLI}}{\mathrm{weight}\;\mathrm{of}\;\mathrm{feeding}\;\mathrm{IR}1048}\times 100\%$$

To quantify the protein profiles of 4T1 cell membrane fragments, sodium dodecyl sulfate polyacrylamide gel electrophoresis (SDS-PAGE) analysis was performed to distinguish well between MLI and LI. In particular, membrane proteins from both 4T1 cell (4T1M) and MLI were measured by using standard BCA Protein Assay Kits (Beyotime Biotechnology, Shanghai, China). After they were prepared with the same protein concentrations, 4T1M, MLI and LI were mixed with loading buffer and heated at 70 °C for 10 min, then run on SDS-PAGE prefabricated gels (Shanghai Yongjing Biotech Co., LTD, Shanghai, China) together with colorful pre-stained protein marker. The gels were stained with Coomassie blue for 30 min, and further washed with pure water overnight and imaged by ChemiDoc™ imaging system (Bio-Rad, Hercules, CA, USA).

### 2.4. In Vitro Photothermal Effect of MLI

The photothermal effect of MLI with the different concentration (7.5, 15, 30, 60 and 120 μM) of IR1048 component was measured upon 1064 nm laser irradiation (1 W/cm^2^) for 6 min when the same volume of pure water was utilized as the control group. The photothermal effect of MLI (30 μM) with different laser powers (0.25, 0.5 and 1 W/cm^2^) was also examined. The temperature changes were recorded by using fluke thermal imager. The photothermal stability of MLI was inspected by measuring the periodic heating process under 1064 nm laser irradiation (1 W/cm^2^) for 6 min followed by the cooling process. The photothermal conversion efficiency (η) of MLI was calculated by using the equation previously reported [28].

### 2.5. Cellular Uptake, Homologous-Targeting Capability and Cytotoxicity of MLI

Three categories of cells 4T1, B16 and Hek293 were used to assess the cellular uptake, homologous-targeting capability and in vitro photothermal cytotoxicity of MLI. In particular, FITC were used to label MLI by encapsulation, DiI and DiO were used to successively label the cell membrane, and Hoechst 33342 was adopted to stain the cell nucleus. In addition, FITC-labeled MLI were incubated with DiI and Hoechst 33342 dual-stained 4T1 cells, and the cellular uptake of MLI was measured at various time points (0 h, 2 h, 4 h, 6 h and 12 h). Subsequently, the cells were washed 3 times with PBS, and then hired flow cytometry was performed to analyze it, whose fluorescence images were captured by a Carl Zeiss LSM 710 laser scanning confocal microscope.

To test the homologous-targeting capability of MLI, Rhodamine B-labeled MLI were incubated with DiO and Hoechst 33342 dual-stained 4T1, B16 and Hek293 cells for 4 h, respectively, and then analyzed by flow cytometer and imaged by a laser scanning confocal microscope. Meanwhile, the in vitro photothermal cytotoxicity of MLI was also inspected. The logarithmic-phase 4T1, B16 and Hek293 cells were respectively seeded into 96-well plates with 7 × 10^3^ cells/each well and grown for 12 h, and then the mediums were replaced by fresh medium containing various concentrations of MLI followed by 6 min 1064 nm laser irradiation. TheCCK-8 staining assay was employed to calculate the relative cell viability, which was measured by a SpectraMax M5 microplate reader (Molecular Devices, San Jose, CA, USA) at 450 nm. Moreover, its cytotoxicity was further assessed by Calcein-AM/PI.

### 2.6. Dual-Modal Photoacoustic and Thermal Imaging-Guided Photothermal Therapy

The in vitro and in vivo photoacoustic imaging (PAI) was to investigate whether the MLI could enhance the photoacoustic effects. The solid phantom made up of 2% ager solution and intralipid embedded with different concentrations of MLI (calculated by IR1048) was immersed into the water. The photoacoustic signals were detected by our home-made PAI system that mainly consists of laser emission system (SURELITE OPO PLUS Optical Parametric Oscillator, Santa Clara, CA, USA), rectangular prism, beam expander, rotator, transducer, water tank, amplifier, trigger line, oscilloscope and computer. The revolving pulsed laser from Nd: YAG (wavelengths: 1064 nm) stimulated the phantom or the mice tumor to generate complex wave field signals that were converted to digital signals after amplification by a Pulse/Receiver (5073R, Olympus, Tokyo, Japan). Then the photoacoustic images were acquired by reconstruction of delay-sum beam forming algorithm. For in vivo tests, the tumor-bearing mice were imaged before and after intravenously tail-injection of MLI under 1064 nm laser irradiation at different time points (0, 2, 4, 8 and 12 h). In addition, the temperature changes of tumor site upon the irradiation of 1064 nm laser were imaged by fluke thermal imager at different time points after tail-vein injection of MLI.

Besides directly recording the temperature of tumor site upon the irradiation of 1064 nm laser by fluke thermal imager at different time points after intravenously tail-injection of MLI. Those tumor-bearing mice were imaged before and after intravenously tail-injection of MLI under 1064 nm laser irradiation at different time points (0, 2, 4, 8 and 12 h) by our home-made multispectral photoacoustic tomography systems (Continuum Surelite I-10; Wavelength: 680~1064 nm; Pulse duration: 5~10 ns; Frequency rate: 20 Hz). Specifically, a pulsed laser from Nd:YAG laser crystal as the laser source illuminated mice tumors through an optical subsystem with 1064 nm wavelength. A 360-degree arc rotation Olympus-NDT V303-SU transducer (10 MHz central frequency; bandwidth range from 0.65 to 1.18 MHz) by a rotary stage was adopted to collect the photoacoustic signals. The emitting complex wave field signals from the mouse tumor were amplified by an Olympus 5073R Pulser/Receiver and then converted into digital signals. Afterwards, the photoacoustic images were gained through reconstruction by a delay-and-sum beam forming algorithm. 

Tumor-bearing mice were used for NIR-II photothermal therapy by tail-vein injections when the tumor size reach to 80~140 mm^3^. The tumor-bearing mice were randomly divided into six groups (n = 5): (1) PBS; (2) MLI; (3) Laser; (4) IR1048 + Laser; (5) LI + Laser; (6) MLI + Laser. The laser irradiation was conducted at 4 h post intravenous injection, and the dose of group 2, 4, 5 and 6 was equivalent to IR1048 of 0.8 mg/kg per mouse, while group 1 and 3 was tail-vein injected with 150 μL PBS as control. Additionally, all the power density of 1064 nm laser irradiation upon the tumor site was 1 W/cm^2^ and the irradiation time was 6 min. The tumor volumes and the body weight were measured every three days using Vernier caliper and electronic scales. As a result, the mice with tumor volumes exceeding 1000 mm^3^ were euthanized. The survival rate of each group was calculated after 30-day post-injection. At last, the mice were sacrificed, and their tumors, heart, liver, spleen, lungs and kidneys were resected for subsequent immunohistochemical analysis stained by hematoxylin and eosin (H&E).

### 2.7. Statistics

All the data were calculated as mean ± standard deviation (S.D.) by GraphPad Prism 7.0. The label of significance was refined by * *p* < 0.05, ** *p* < 0.01, *** *p* < 0.001 and **** *p* < 0.0001.

## 3. Results and Discussion

### 3.1. Preparation and Characterization of MLI

**Figure 1:**
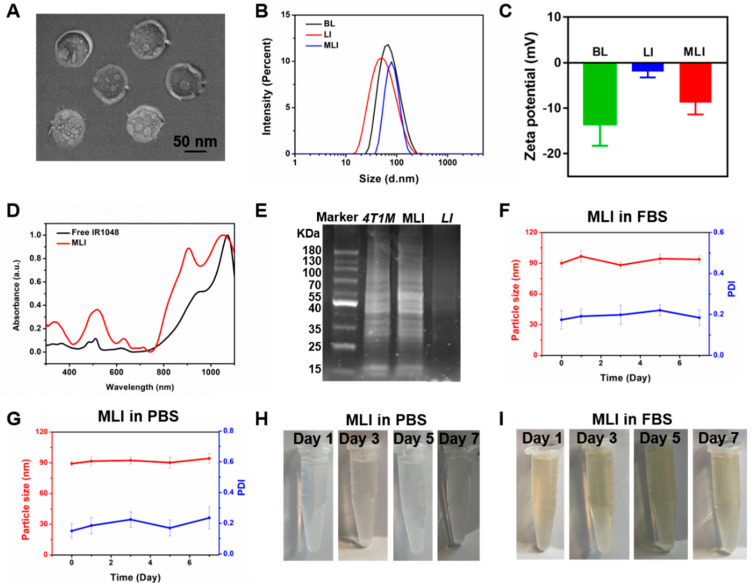
Characterization of MLI. (**A**) TEM image of 4T1 cell membrane-camouflaged IR1048-loaded liposome (MLI). (**B**) Particle size distribution of blank liposome (BL), IR1048-loaded liposomes (LI) and MLI, respectively. (**C**) Zeta potentials of BL, LI and MLI, respectively. (**D**) UV–vis–NIR absorption spectra of free IR1048 and MLI. (**E**) SDS-PAGE analysis of protein marker, 4T1 cell membrane proteins, MLI and LI solutions, respectively. Particle sizes (red curve) and PDI (blue curve) variations of MLI (**F**) in fetal bovine serum (FBS) and (**G**) in phosphate buffer solution (PBS, pH = 7.4) in 7 days. MLI solution photographs in (**H**) PBS (pH = 7.4) and in (**I**) FBS at various time.

The overall design idea of this study is that the MLI in short of 4T1 mouse breast cancer cell membrane fragments coated IR1048-loaded liposomes, held the potential by homologous-targeting to 4T1 breast cancer for enhancing photothermal therapy in the second near-infrared window (NIR-II) under the photoacoustic and thermal dual-modal imaging guidance. Specifically, the MLI was prepared by film dispersion and membrane extrusion method, and their morphology and particle size were characterized using transmission electron microscopy (TEM) and dynamic light scattering (DLS) measurements, in which MLI exhibited uniform spherical morphology (Figure 1A) with an average diameter of 92.55 ± 5.41 nm (Figure 1B). MLI were uniformly dispersed and camouflaged by chiseled monolayer adventitial coat and analogous proteins that were negative staining by tungsten phosphate. It was interested that the particle size of blank liposome (for short: BL) distributed in 95.61 ± 5.83 nm got larger after encapsulating IR1048 dye into liposomes (for short: LI) with size of 98.26 ± 4.76 nm, but turned smaller after coating with 4T1 cell membrane fragments, indicating that the membrane fragments made the liposomes more compacted during extrusion. Changes also happened on their zeta potential (Figure 1C), in which the BL with −13.58 mV became −1.94 mV after loading IR1048, and then turned to −8.80 mV.

**Figure 2:**
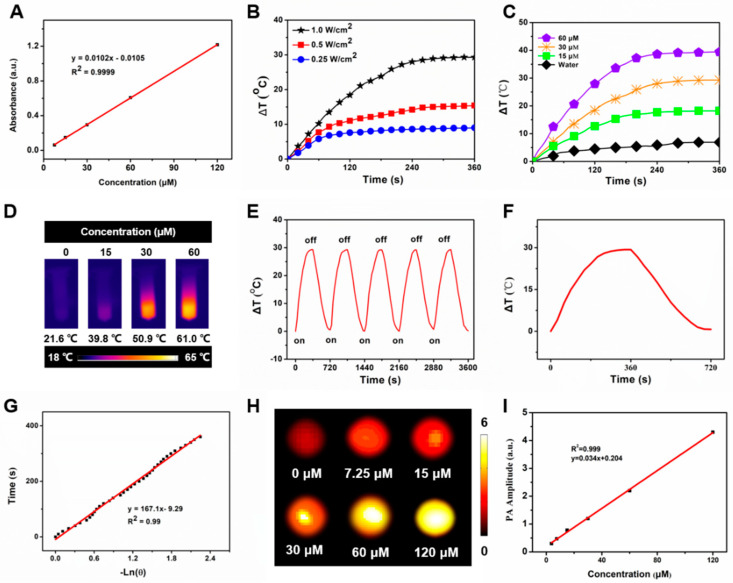
In vitro photothermal performance of MLI. (**A**) Standard curve of IR1048. (**B**) Temperature behavior of MLI upon various 1064 nm laser powers (1.0, 0.5 and 0.25 W/cm^2^). (**C**) Temperature evolution curves of MLI with various concentrations (IR1048 equivalent to 0, 15, 30 and 60 μM) upon 1064 nm excitation. (**D**) Thermal images of MLI solution with various concentrations (IR1048 equivalent to 0, 15, 30 and 60 μM) upon 1064 nm laser irradiation. (**E**) Temperature variation of MLI through 5 laser on/off cycles. (**F**) Photothermal behavior of MLI during heating and cooling period. (**G**) Linear correlation between the time and −lnθ calculated from the cooling database. (**H**) In vitro PAI with various concentrations of MLI (IR1048 equivalent to 0, 7.25, 15, 30, 60 and 120 μM). (**I**) Linear relationship between the concentrations of MLI and their PA amplitudes under 1064 nm laser excitation.

What is more, it showed that the free IR1048 possess a characteristic absorption band around 1071 nm, and MLI also presented a similar NIR-II absorption peak in the UV–vis–NIR absorption spectra (Figure 1D). Hence 1064 nm continuous wave laser was employed to explore their photothermal performance. Further, to analyze the protein profile of MLI, SDS-PAGE analysis was performed. Interestingly, similar protein profiles were detected for both the MLI and 4T1 membrane (Figure 1E). However, this is not the case for LI, demonstrating that MLI was successfully coated by 4T1 membrane fragments. In addition, to assess the stability of MLI, the particle sizes of MLI either in PBS or fetal bovine serum (FBS) were continuously tested at 37 °C for one week. The results showed that their average size and polymer dispersity index (PDI) were very stable with only slight fluctuation (Figure 1F–I). Moreover, MLI presented a homogeneous solution with milk light, indicating that MLI significantly improved the poor water-solubility of IR1048 organic dye. According to the standard curves associated with IR1048 dye (Figure 2A), the loading efficiency of IR1048 dye and its encapsulation efficiency in MLI was 18.4% and 94.7%, respectively. Taken together, the above characterization conformed the successful preparation of MLI.

### 3.2. In Vitro NIR-II Photothermal Performance and PAI Capability

The temperature profiles were real-time recorded by an infrared thermal imager. Among 0.25, 0.5 and 1.0 W/cm^2^ of 1064 nm laser used to irradicate 30 μM MLI, only 1.0 W/cm^2^ could rapidly elevated its temperature from 21.6 °C to 50.9 °C, namely its temperature raise was up to 29.3 °C (Figure 2B), which met the requirement of cancer photothermal therapy that it should be higher than 45 °C to ablate the tumors, indicating that laser power significantly affected the photothermal performance of MLI and 1 W/cm^2^ was the best one for MLI-based PTT. In addition, MLI suspension exhibited rapid temperature rise up to 39.4 °C by a concentration-dependent way (15, 30 and 60 μM equivalent to IR1048) upon six-minute 1 W/cm^2^ 1064 nm laser excitation, whereas the temperature of H_2_O only elevated slightly around 6.9 °C (Figure 2C,D). After five laser-on and off cycles upon 1 W/cm^2^ 1064 nm laser irradiation, the MLI still showed similar temperature profiles, demonstrating their excellent photostability (Figure 2E). From the cooling period of MLI (Figure 2F), the photothermal conversion coefficient of MLI was calculated to be 36.53% (Figure 2G) In addition, MLI exhibited excellent NIR-II PAI capabilities (Figure 2H). In particular, the phantom test results demonstrated a linear correlation between the PA signal intensities and the concentration of MLI (Figure 2I).

### 3.3. Homologous-Targeting Capability, Cellular Uptake and Cytotoxicity of MLI

Nanoplatforms with superior tumor-targeting capability played a vital role in enhancing the selectivity and decreasing non-targeting adverse effects of PTT. In addition, high cellular uptake was associated with possible cellular cytotoxicity and high tumor accumulation. In particular, to examine the cellular uptake of MLI, FITC-labeled MLI were incubated with DiI and Hoechst 33342 dual-stained 4T1 cells for 0 h, 2 h, 4 h, 6 h and 12 h, respectively. It turned out that the mean fluorescent intensity of intracellular MLI exhibited the highest values for 4 h incubation (Figure S1). Therefore, 4-h coincubation of Rhodamine B-labelled MLI or LI (red) with different DiO/Hoechst 33342 dual-stained cells (green and blue) was applied to inspect the homologous-targeting ability of MLI. The corresponding Z-stack confocal images of MLI and LI in Figures S2 and S3, respectively, validating the homologous-targeting capability of MLI.

**Figure 3:**
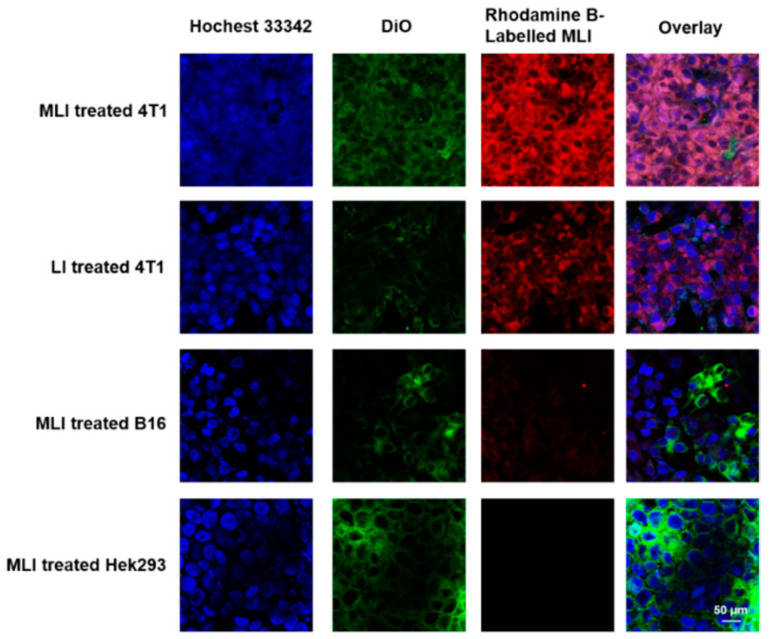
In vitro inspection of homologous-targeting capabilities of MLI. Intracellular co-localization of Rhodamine B-labelled MLI or LI (red) to various DiO/Hoechst 33342 dual-stained cells (green and blue) after 4 h co-incubation.

**Figure 4:**
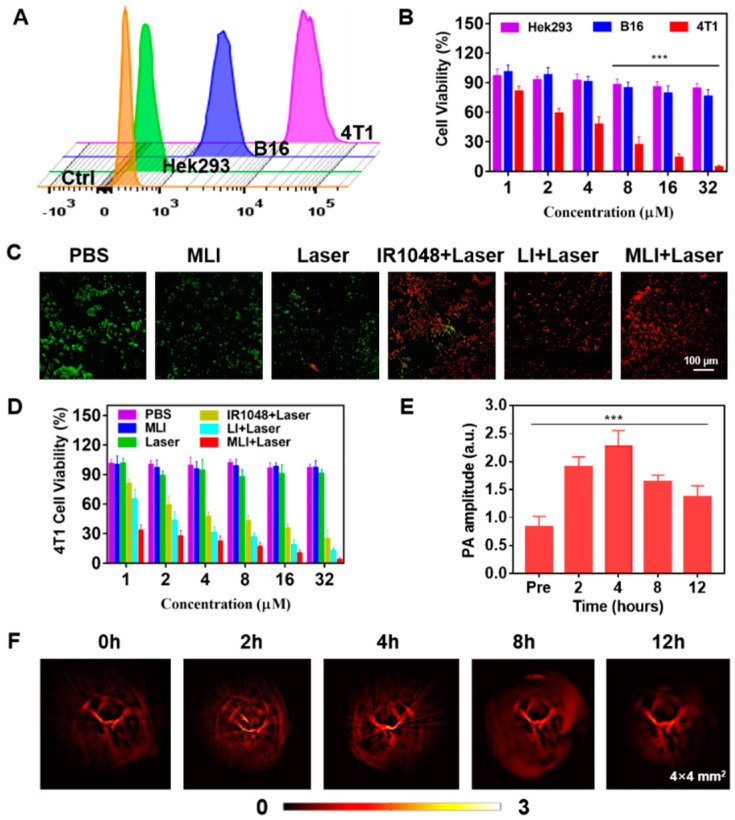
Cellular uptake, cytotoxicity and in vivo photoacoustic imaging. (**A**) Fluorescence intensity of MLI after incubation with various categories of cells for 4 h by flow cytometry. (**B**) Cytotoxicity investigation after treated with MLI with different concentrations upon 6 min 1064 nm laser irradiation. (**C**) Calcein-AM/PI (red: dead cells; green: live cells) dual-stained fluorescence images of 4T1 cells with various treatments. (**D**) 4T1 cell viability after different treatments with various concentrations. (**E**) In vivo PA amplitude variation at various time. (**F**) PAI of tumor vasculatures at different time points before and after tail-vein injection of MLI. The PAI imaging regions were 4 × 4 mm^2^. *** *p* < 0.001.

In addition, it was discovered that the cellular uptake of MLI by breast cancer 4T1 cells was much higher than that by the melanoma B16 cells and human embryonic kidney Hek293 cells. In addition, the cellular uptake of MLI by 4T1 cells was also much higher than the uptake of LI (Figure 3), implying that 4T1 cell membrane fragments coated MLI was able to enhance the cellular uptake ability. More importantly, flow cytometry analysis results revealed that cellular uptake of MLI was much higher by 4T1 cancer cells as compared to those from the B16 cells and Hek293 normal cells (Figure 4A). Further, the standard CCK-8 assay was utilized to assess the photothermal cytotoxicity of MLI to the three categories of cells (Figure 4B). It was discovered that MLI was able to kill over 95.49% 4T1 cells after 6-min laser (1064 nm) irradiation. However, MLI with laser can only eliminate less than 23.85% B16 cells and 15.69% Hek293 cell, respectively. These findings demonstrated that MLI can significantly improve the selectivity and specificity of PTT at the cellular level. Interestingly, the confocal imaging results in Figure 4C illustrated the 4T1 tumor cells treatment efficacy by various strategies. Then, as plotted in Figure 4D, PBS, MLI and laser alone hardly cause 4T1 cell death, whereas the LI + laser and MLI + laser groups could kill up to 87.24% and 96.16% of 4T1 cells. Hence, these results demonstrated that MLI exerted the strongest cytotoxicity to the homologous cancer cells owing to the homologous-targeting ability endowed by the same cancer cell membrane, and it barely showed toxicity to normal cells which is important to targeted cancer therapy. 

### 3.4. Dual-Modal Photoacoustic and Photothermal Imaging Guided NIR-II PTT In Vivo

4T1 tumor-bearing BALB/c nude mice model was established to inspect the dual-modal NIR-II photoacoustic and photothermal imaging ability of MLI. After tail-vein injection of MLI, the tumor vascular structures were imaged and monitored at various time points. The photoacoustic signals were acquired at 0 h, 2 h, 4 h, 8 h and 12 h by using our home-made PAI system with 1064 nm pulsed laser illumination. It was discovered that enhanced photoacoustic signals were detected after the injection of MLI largely due to the tumor-targeted accumulation of MLI (Figure 4E). In addition, the PA signals exhibited the most significantly contrast enhancement at 4 h post-injection although the signal strength gradually decreased after 4 h, implying that 4 h post tail-vein injection was the best time to carry out PTT (Figure 4F). In addition to NIR-II PAI, photothermal imaging was also performed to guide PTT. 

**Figure 5:**
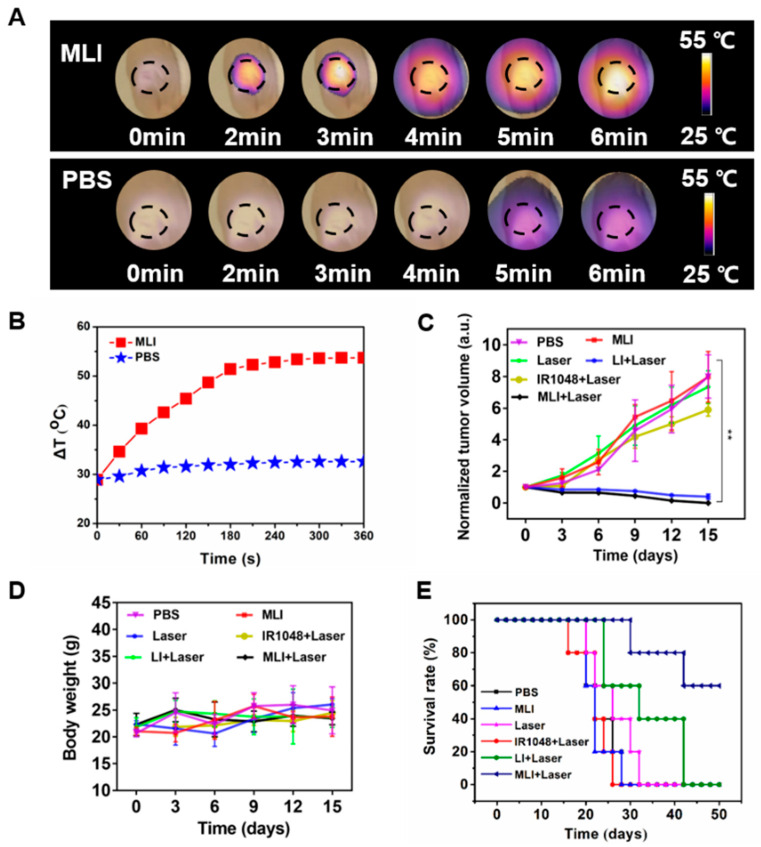
Thermal recording and anti-cancer evaluation of MLI. (**A**) In vivo photothermal images of tumors after intravenous injection of MLI and PBS upon 1064 nm laser excitation. (**B**) Temperature profiles of tumor site after tail-vein injection of MLI and PBS. (**C**) Normalized tumor volume variations. (**D**) Body weight variations of different treatment groups. (**E**) Mice survival curves for various treatment groups. ** *p* < 0.01.

**Figure 6:**
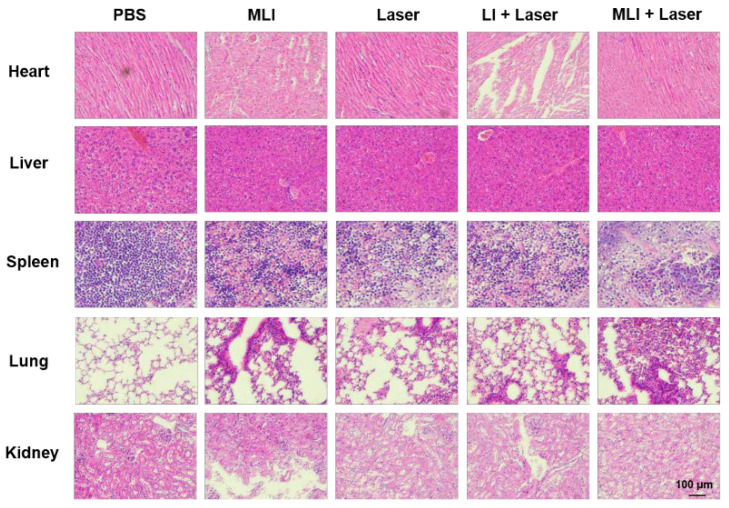
Representative H&E-stained images of the major organs (heart, liver, spleen, lung and kidney) extracted from mice with different treatment groups. (Scale bars: 100 μm).

Meanwhile, the efficacy of NIR-II PTT in vivo was carried out at 4 h post tail-vein injection of MLI for 6 min with 1 W/cm^2^ power of 1064 laser, during which the temperature changes of tumor site were carefully monitored by photothermal imaging (Figure 5A). Specifically, the 4T1 tumor-bearing nude mice were randomly divided into five treatment groups: (i) phosphate buffered saline (PBS) only, (ii) MLI only, (iii) PBS with laser, (iv) LI with laser and (v) MLI with laser. Interestingly, compared to the control groups of PBS or PBS + laser, the MLI + laser treatment group was able to quickly elevate the tumor-site temperature up to 53.7 °C in 6 min (Figure 5B). In addition, Figure 5C demonstrated that MLI + laser group exhibited significant capability to inhibit tumor growth, which almost ablated the whole tumors with slight body weight variation (Figure 5D). Interestingly, it is not significant difference in the tumor volumes growth inhibition between MLI + laser and LI + laser groups, which was probably due to the fact that LI might be able to reach the tumor site similarly to MLI, because the liposomes itself mimics the cell membrane and could be uptaked into the cancer cells by membrane fusion. Both of which depend on the photothermal effect of IR1048 dye to ablate the tumors. While the survival curves also indicated that the MLI + laser group possessed the highest average life span over the therapy episode as compared to those from other treatment groups (Figure 5E). To further assess the systemic toxicity of MLI, the hematoxylin and eosin (H&E) staining was performed for most of the extracted organs such as heart, lung, kidney, liver and spleen after 30-day treatments. The H&E analysis results (Figure 6) suggested that the MLI caused no observable damage to the normal organs.

## 4. Conclusions

The developed MLI has been demonstrated to be an eligible tumor-targeting nanotheranostic due to 4T1 cell membrane coating endowed tumor homologous-targeting. Meanwhile, it not only emerged the capacity to be the contrast agent of photoacoustic and thermal imaging to noninvasively monitor the tumor-accumulation amounts and metabolization speed through the enhanced PA amplitudes of MLI, but also could serve as an excellent NIR-II photothermal agent to accurately ablate the solid tumors with awesome efficacy in vivo. Furthermore, the uniform spherical MLI preparation extremely improved the hydrophobicity of the NIR-II organic dye IR1048, paving the way for its broaden biomedical applications. Moreover, MLI was the most uptaked by 4T1 cells after 4-h co-incubation among other type of cells and showed the strongest cytotoxicity to 4T1 cells with photothermal conversion efficiency of 36.53%. Having worked out the positive correlation between PA intensity of MLI and its concentration, we realized accurate track-down of the MLI in vivo and the temperature of photothermal therapy by thermo-imaging. In the tumor-bearing mice model, MLI obviously enhanced the in-vivo imaging effect of photoacoustic tomography, in which the tumor vasculature variation and the retention time of MLI in tumors were intuitively observed. Therefore, under the guidance of NIR-II photoacoustic imaging (1064 nm pulsed laser) and thermo-imaging, MLI was evidenced to be high-efficient therapeutics in eliminating breast cancers upon NIR-II continuous laser with minimal adverse-effect to other organs thanks to the specifical homologous tumor-targeting ability endowed by congener cancer cell membrane disguise. Hence, the MLI was a representative “one for all” multifunctional nano-system to treat breast cancer.

## Supplementary Materials

The following supporting information can be downloaded at: https://www.mdpi.com/article/10.3390/pharmaceutics14071367/s1, Figure S1. Cellular uptake of MLI to 4T1 cells at various incubation time. From left to right, Hoechst 33342-stained cell nuclei, FITC-labelled MLI, DiI-stained cell membrane and overlay images, respectively. All scale bars correspond to 25 μm. Figure S2. Z-stack confocal images of the Rhodamine B labeled LI, Hoechst 33342 and DiO dual-stained 4T1 cells. All scale bar corresponds to 50 μm. Figure S3. Z-stack confocal images of the Rhodamine B labeled MLI, Hoechst 33342 and DiO dual-stained 4T1 cells. All scale bar corresponds to 50 μm.
