# Supplementary material for: Breast Cancer Cell Membrane Camouflaged Lipid Nanoparticles for Tumor-Targeted NIR-II Phototheranostics
Source: Pharmaceutics. 2022 Jun 28;14(7):1367. doi: 10.3390/pharmaceutics14071367 (PMC9319009; doi:10.3390/pharmaceutics14071367)
Supplement: Supplementary file 1 [file pharmaceutics-14-01367-s001.zip › pharmaceutics-1746399-supplementary.pdf]

Supporting Information

Breast Cancer Cell Membrane Camouflaged Nanoparticles for Homologous-targeted NIR-II Phototheranostics

Mengze Xu[1,2], Yu Yang[3], Zhen Yuan[1,2,*]

[1] *Cancer Center, Faculty of Health Sciences, University of Macau, Macau 999078, P. R. China*

[2] *Centre for Cognitive and Brain Sciences, University of Macau, Macau 999078, China*

[3] *Institute of Molecular Medicine (IMM), Renji Hospital, Shanghai Jiao Tong University School of Medicine, and College of Chemistry and Chemical Engineering, Shanghai Jiao Tong University, Shanghai 200240, China*

* Corresponding author: E-mail: zhenyuan@um.edu.mo, Phone: +00 8538822 4989, Fax: +00 853 8822 2314

The photothermal conversion efficiency (η) was calculated according to the Equation S1[Ref:1]：

$$\eta = \frac{hs(T_{max} - T_{surr}) - Q_{Dis}}{I(1 - 10^{-A1064})} \quad (S1)$$

Where h means the heat transfer coefficient, s represents the surface area of the container, T_{max} is equilibrium temperature, T_{surr} is the ambient temperature of the environment, $Q_{Dis}$is the heat dissipation from the light absorbed by the solvent and the container, I is the incident laser power density, A1064 represents the absorption of sample at 1064 nm. The calculation of the hs is following the Equation (S2).

$$\tau_s = \frac{m_D c_D}{\hbar s} \quad (S2)$$

Where τ_s means the time constant for heat transfer of the system, m_D and c_D represents the mass and heat capacity (4.2 $J{\cdot}g^{-1}{\cdot}℃^{-1}$) of the solvent, respectively. The value of τ_s is calculated according to the Equation (S3) and (S4).)

$$t = -\tau_s \ln \theta \quad (S3)$$

$$\theta = \frac{T - T_{surr}}{T_{max} - T_{surr}} \quad (S4)$$

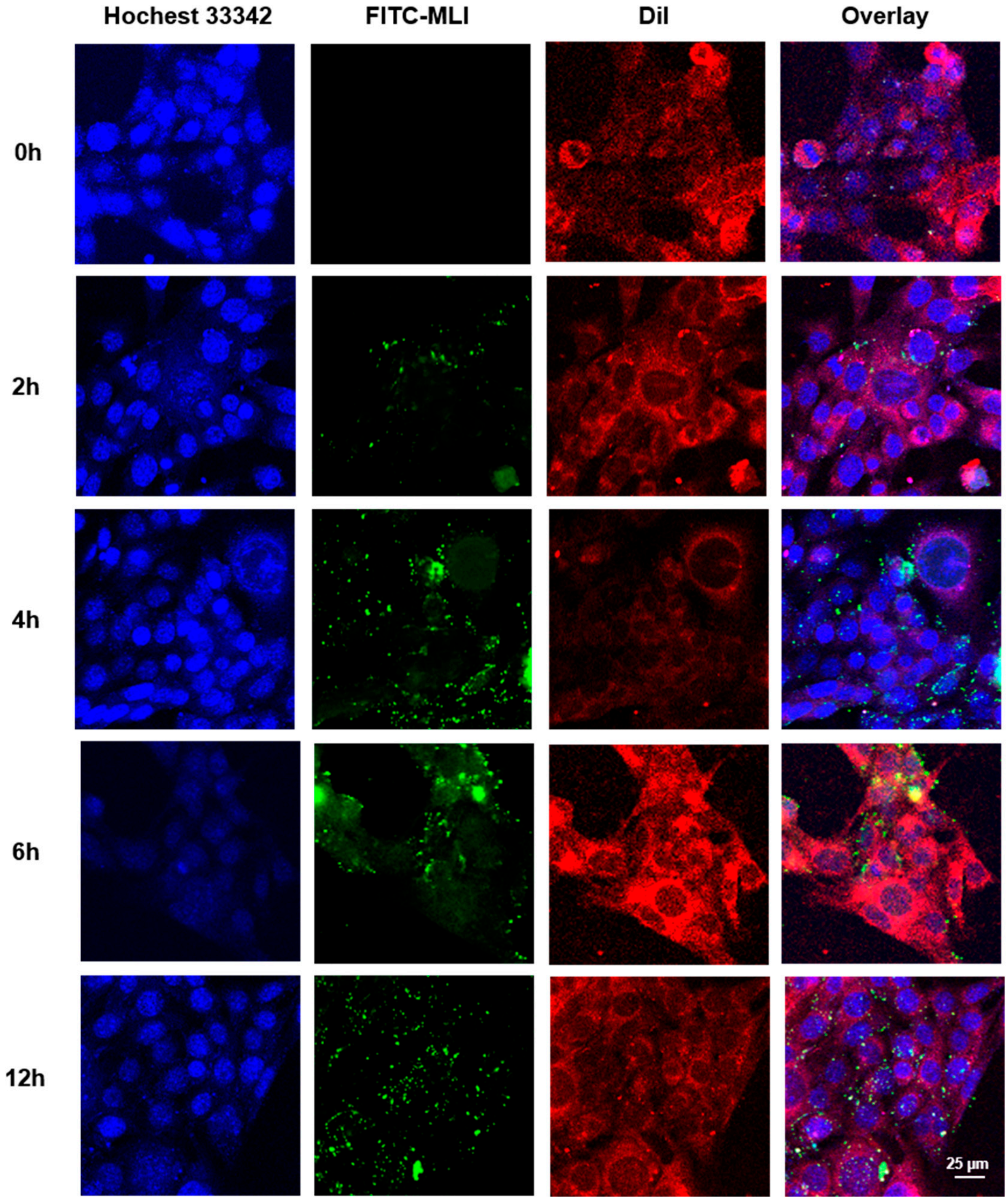

Figure S1. Cellular uptake of MLI to 4T1 cells at various incubation time. From left to right, Hoechst 33342-stained cell nuclei, FITC-labelled MLI, DiI-stained cell membrane and overlay images, respectively. All scale bars correspond to 25 μm.

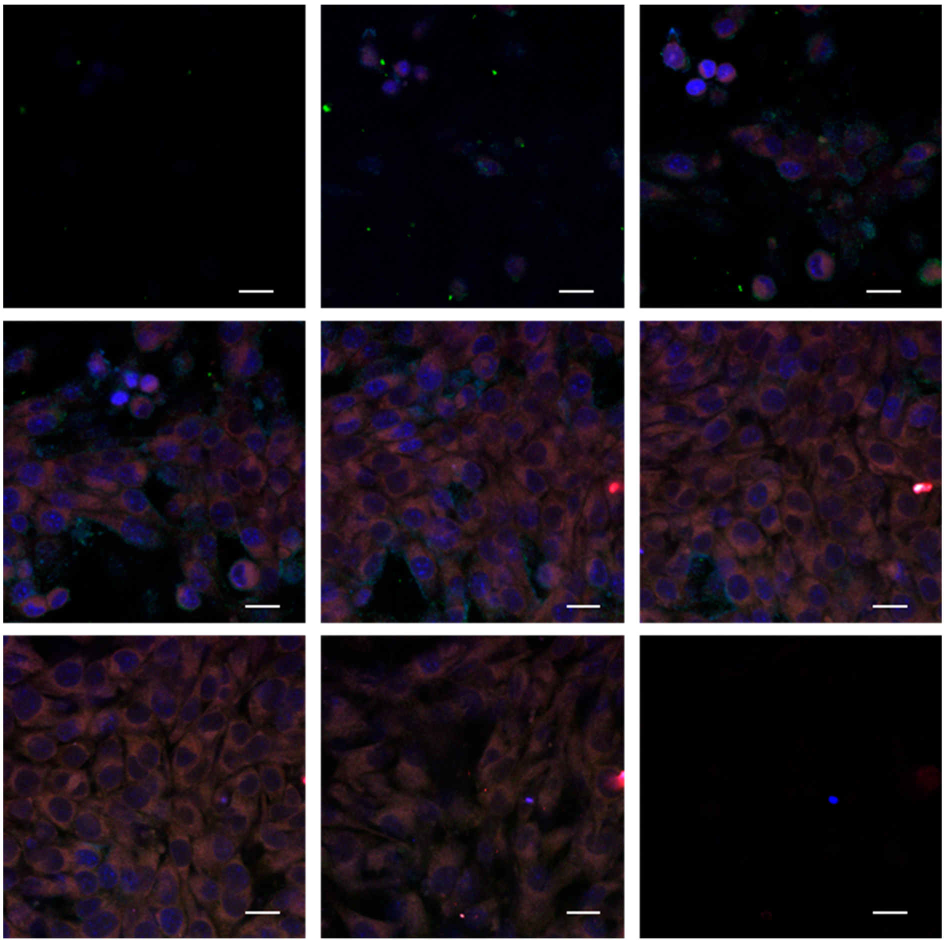

Figure S2. Z-stack confocal images of the Rhodamine B labeled LI, Hoechst 33342 and DiO dual-stained 4T1 cells. All scale bar corresponds to 50 μm.

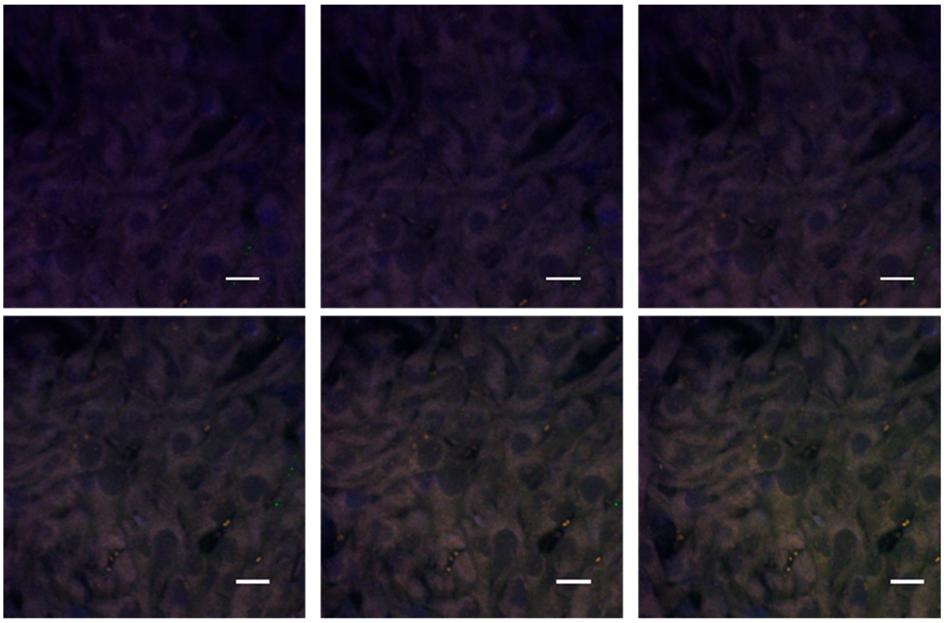

Figure S3. Z-stack confocal images of the Rhodamine B labeled MLI, Hoechst 33342 and DiO dual-stained 4T1 cells. All scale bar corresponds to 50 μm.

Reference:

[1] Chen Z, Zhang Q, Zeng L, Zhang J, Liu Z, Zhang M, Zhang X, Xu H, Song H, Tao C. Light-triggered OVA release based on CuS@poly(lactide-co-glycolide acid) nanoparticles for synergistic photothermal-immunotherapy of tumor. Pharmacol Res. 2020 Aug; 158: 104902. doi: 10.1016/j.phrs.2020.104902.
